# Analysis of Anatomical pathology laboratory capabilities in eastern Democratic Republic of Congo

**DOI:** 10.1371/journal.pgph.0006067

**Published:** 2026-03-18

**Authors:** Tshass Chasinga Baharanyi, Rodrigue Ayagirwe Basengere, Jonathan Yoyu Tunangoya, Marie Hallin, Baudouin Manwa Budwaga, Espoir Bagula Mukengere, Ghislain Bisimwa Balaluka, Denis Mukwege Mukengere, Zisis Kozlakidis, Bienvenue Lebwaze, Olivier Vandenberg

**Affiliations:** 1 Faculty of Medicine, Université Evangélique en Afrique, Bukavu, Democratic Republic of the Congo; 2 Centre for environmental and occupational health, School of Public Health, Université libre de Bruxelles, Brussels, Belgium; 3 Molecular Biology Laboratory, Université Evangélique en Afrique, Bukavu, Democratic Republic of the Congo; 4 International Center for Advanced Research and Training, Panzi Foundation, Bukavu, Democratic Republic of Congo; 5 European Plotkin institute for vaccinology (EPIV), Faculty of medicine, Université libre de Bruxelles, Brussels, Belgium; 6 Biomedical Laboratory, Official University of Bukavu, Bukavu, Democratic Republic of the Congo; 7 Regional School of Public Health and School of Medicine, Université Catholique de Bukavu, Bukavu, Democratic Republic of Congo; 8 Centre de Recherche en Sciences Naturelles, Lwiro, Democratic Republic of Congo; 9 Gynecology and General Surgery, Panzi General Referral Hospital, Bukavu, Democratic Republic of the Congo; 10 Laboratory Services and Biobanking, International Agency for Research on Cancer, World Health Organization, Lyon, France; 11 Division of Pathology, University Hospital of Kinshasa, School of Medicine, University of Kinshasa, Kinshasa, Democratic Republic of Congo; 12 Research and Technology Innovation Unit, Laboratoire Hospitalier Universitaire de Bruxelles (LHUB-ULB), Brussels, Belgium; 13 Division of Infection and Immunity, Faculty of Medical Sciences, University College London, London, United Kingdom; PLOS: Public Library of Science, UNITED STATES OF AMERICA

## Abstract

The objective of this study was to evaluate the infrastructure and diagnostic capacity of pathology laboratories in eastern Democratic Republic of Congo (DRC). A multi-site study was conducted using a mixed methods approach to analyse laboratory equipment, human resources and diagnostic performance. Data were collected from February to April 2023 in eastern DRC. The results show a concentration of laboratories in urban areas covering a population of 3 provinces (South Kivu, North Kivu and Haut Katanga), leaving 9 provinces with no diagnostic coverage and a population of over 26.8 million people. The analysis revealed a significant gap in anatomical pathology laboratory services. Major deficiencies were identified, including the lack of immunohistochemistry, which is only available at the Panzi General Referral Hospital in South Kivu Province, the absence of computerised sample tracking systems and non-standardised quality control protocols. Human resources are also insufficient, with most laboratories operating with a single pathologist and minimal histotechnician support. Histopathology accounted for 73.4% of processed samples, with inflammatory and infectious lesions comprising 41.2% of diagnoses. The most common malignancies were cervical cancer (16.6%), prostate cancer (14.1%), breast cancer (13.9%), and colorectal cancer (3.0%). Limited cytological analyses, particularly the absence of fine-needle aspiration procedures, further hinder diagnostic accuracy. These findings underscore the urgent need to expand and equip pathology laboratories, implement standardized quality assurance measures, and establish continuous training programs for laboratory personnel. Addressing these deficiencies is critical to improving cancer diagnostics and broader healthcare services in the DRC.

## Introduction

Cancer is a global public health issue affecting all categories of the world’s population [1]. In sub-Saharan Africa, cancer incidence estimates are still patchy, as many countries do not have reliable systems for collecting health data [2]. However, recent regional estimations demonstrate that without rapid interventions, a major increase in cancer mortality should be anticipated, from 520,348 in 2020 to about 1 million deaths per year by 2030 [3]. Thus, existing healthcare systems would need to plan for accommodating this increase across their populations – and consider the centrality of laboratory-based pathology in the detection and treatment of cancer [4].

Laboratory services are essential to provide adequate and equitable medical care; it is estimated that at least 70% of medical decisions (particularly those involving accurate diagnosis) in high-income countries depend on laboratory data [5]. At the same time, pathology services, which are recognized as essential for the diagnosis and choice of treatment for cancer, are poorly developed in sub-Saharan countries. This situation is exacerbated by significant structural challenges within health systems, including shortages of qualified personnel and financial resources, as well as inadequately equipped laboratory infrastructure [5,6].

The Democratic Republic of Congo (DRC), located in the centre of Africa, spans an area of 2,345,000 km², making it the second largest country on the continent. Extending from 5°23′N to 13°26′S and from 12°12′E to 31°17′E, it is nearly as large as Western Europe. For instance, its surface area is approximately five times that of France, which is 551,695 km². Additionally, the DRC is slightly larger than the half of European Union, which has a total area of 4,233,255 km². The DRC is the fourth most populous country in Africa, with a population exceeding 100 million in 2020 [7]**.** Comprising 25 provinces, the DRC represents one of the most challenging environments for health development in sub-Saharan Africa [8–10]. The climate in the DRC is hot and humid, with an annual average temperature of 25°C, except in the south-eastern highlands [11].

The DRC has experienced recurrent instability for more than three decades, and the consequences for an already fragile healthcare system have been devastating [12]. The eastern region of the country faces significant challenges, including political unrest, socio-economic disparities, disparities in access to healthcare and under-funding of physical and healthcare infrastructure. This situation is being exacerbated by fragile security conditions, poor governance and economic mismanagement [13,14].

There is a lack of detailed historical data on laboratory capacity in the DRC. However, capacity-building projects funded by USAID, Fondation Mérieux, DIFAEM, Pain pour le Monde and others have been launched in recent years, which unfortunately were limited to big towns. [15,16].

Therefore, in this context, the provision of essential services such as healthcare remains profoundly affected, including in cancer diagnosis and treatment [4,17]. Moreover, while the incidence of cancer on the continent continues to rise, the tools needed to collect data, such as the cancer register, which depends mostly on laboratory diagnosis, are not functioning, hampering efforts to identify and combat cancer [4]. In DRC, the incidence of cancer is 23,033 in a population of 47,559,978 men. It is 29,033 for 47,680,804 women and 52,066 for a population of 95,240,782 for both sexes combined. In men, the most common cancers are prostate, liver and colorectal; in women, cervical cancer is the most common, followed by breast cancer and colorectal cancer in third place. The most common cancers in the global population are, cervical, prostate, breast and colorectal [18].

As a preamble to addressing these challenges, it is necessary to understand the current state of pathology laboratory infrastructure in DRC and its geographical distribution. This research aims to assess the infrastructure capacity, the operational system and human resources capacity of anatomical pathology laboratories in eastern DRC, identify gaps and bottlenecks, develop recommendations and propose strategies based on local data to strengthen diagnostic capacity, by analyzing quantitative data on laboratory activities and qualitative insights into infrastructure, human resources, diagnostic processes, and systemic challenges..

## Methods

### Study area

The survey took place from February 1 to April 28, 2023 and covered eleven provinces in the eastern region of the Democratic Republic of Congo (DRC), namely Haut Katanga, Lomami, Haut-Lomami, Tanganyika, Lualaba, Maniema, South Kivu, North Kivu, Ituri, Tshopo, and Haut-Uélé. These provinces of eastern DRC are a vast and diverse region with varying levels of population density, health infrastructure and security. It is divided into health zones, with urban health centres generally having better equipped medical facilities. However, numerous rural health zones continue to experience limited access to essential health services, particularly pathology laboratory services. Given the complexity of the socio-economic and political environment, health services in these regions are often affected by limited resources, logistical constraints and conflict. Therefore, the first stage identified the provinces with at least one anatomical pathology laboratory. Subsequently, the structure, functioning, and results of each laboratory were analyzed in detail in each province.

### Study design

This study used a mixed methods approach, integrating quantitative data from laboratory records and qualitative insights from key informant interviews. The Donabedian framework was used to assess the structure, processes and outcomes of anatomical pathology laboratory in eastern DRC. This model, widely used in healthcare quality assessment, allows for a systematic assessment of diagnostic capacity and highlights critical gaps in laboratory infrastructure and service delivery [19,20]**.**

### Sampling strategy

Given the limited number of pathology laboratories, an exhaustive sampling method was used, including all identified laboratories in South Kivu, North Kivu, and Haut-Katanga. Regarding the collection of quantitative data, the sampling was exhaustive and non-probabilistic. Non-probabilistic purposive sampling was applied for qualitative data collection, targeting laboratory directors, pathologists, technicians, and provincial health officials to ensure a comprehensive understanding of operational challenges [21,22].

### Data collection and analysis

#### Quantitative data collection.

Quantitative data were obtained from cytology and histology registries across the identified pathology laboratories in South Kivu, North Kivu, and Haut-Katanga. These records provided essential information on laboratory statistics, including the average number of samples received per year, the proportion of samples collected from admitted patients versus external sources, and the types of analyses conducted. The study also documented the origin of samples, distinguishing between those received from the province hosting the laboratory, provinces without pathology services, and foreign countries.

The distribution of samples across hospital departments was analyzed, particularly those originating from the Department of Internal Medicine, Department of Surgery, Department of Gynecology and Obstetrics, and Department of Pediatrics. The study further categorized the types of pathological analyses, differentiating between histology and cytology. For histological analyses, data were collected on simple biopsies, exeresis procedures, and partial or total organ resections. Diagnostic outcomes were classified into normal cases, inflammatory or infectious lesions, various pathological conditions, benign tumors, precancerous lesions, and malignant tumors. Additionally, a detailed assessment of malignant tumor locations was conducted, focusing on cancers affecting the breast, cervix, prostate, lymphoid tissue, skin, colorectal region, and other anatomical sites. These data provided a quantitative foundation to assess the operational capacity of each facility, allowing for comparisons between provinces and highlighting disparities in pathology service provision.

#### Qualitative data collection.

In addition to the quantitative analysis, semi-structured interviews were conducted with a range of key stakeholders including laboratory director, pathologist, technician and provincial health official. Participants were selected using predefined criteria, including professional role, relevant experience in anatomical pathology services, availability and consent of participating in the study. Where respondent was less than two per category, they were all interviewed, however when there were more than two, a selection of two was performed. This approach ensured the inclusion of individuals directly involved in or knowledgeable about laboratory operations. The aim was to capture a comprehensive understanding of the infrastructure, human resources, diagnostic protocols, and operational challenges of anatomical pathology laboratories. Interview guides focused on the availability of essential equipment, standard operating procedures, staff training, and financial constraints. Thematic analysis was applied to the interview data to identify patterns related to quality control practices, diagnostic standardization, and logistics for sample handling. This qualitative component was crucial for contextualizing the quantitative findings and for revealing systemic factors that affect anatomical pathology services in the eastern provinces of the Democratic Republic of Congo [20].

### Statistical data analysis

Content analysis was applied iteratively to examine qualitative data, directly incorporating observations made during data collection, including during transcription, until thematic saturation was reached. In parallel, quantitative data were analysed using descriptive statistics to describe laboratory outcomes in eastern DRC. Data analyses were triangulated by integrating quantitative analyses with qualitative findings and cross-validating results through systematic comparison of convergent and divergent patterns across data sources. It is not obligatory for the study to comply with the CONSORT 2010 guidelines, given that it does not constitute a clinical trial or experimental study.

### Ethical considerations

Ethical approval for this study was obtained from the National Ethics Committee and registered under reference number CNES 001/DPSK/209PP/2022 prior to the commencement of data collection. Written informed consent was obtained from participants prior to conducting interviews. For retrospective data collection, consent was not required. Throughout the research process, confidentiality and anonymity were strictly maintained

## Results

### Overview of laboratory infrastructure

**Fig 1:**
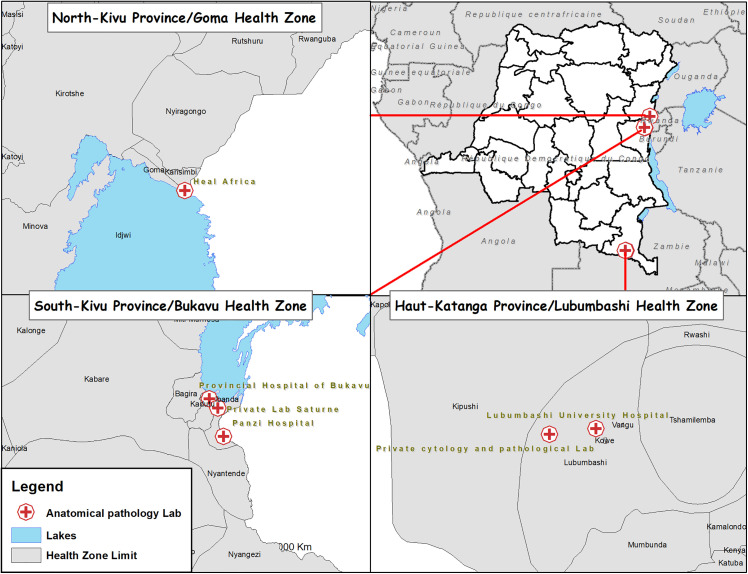
Anatomical Pathology laboratories names and locations in eastern part of DR Congo. The Administrative boundary was accessed through the Référentiel Géographique Commun (RGC) public domain of DR Congo, https://www.rgc.cd/.

**Fig 2:**
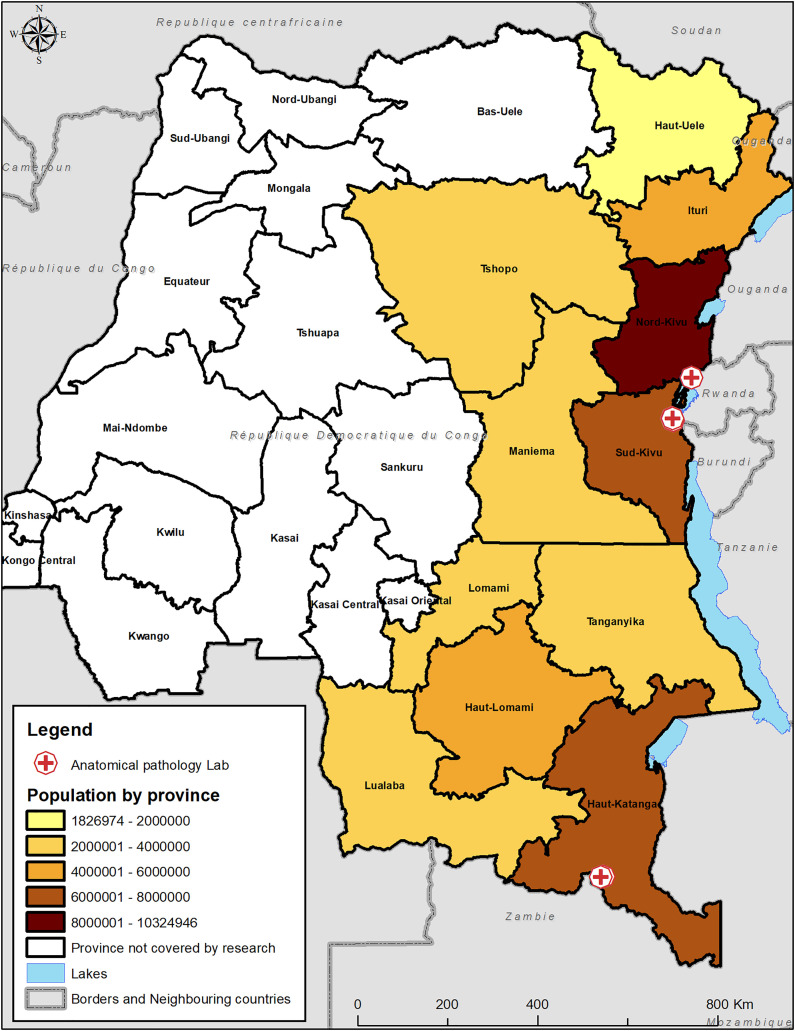
Anatomical Pathology Lab locations eastern part of DR Congo with health province covered. The Administrative boundary was accessed through the Référentiel Géographique Commun (RGC) public domain of DR Congo, https://www.rgc.cd/.

Our survey revealed the presence of six laboratories capable of making a histological and/or cytological diagnosis, distributed across the eleven eastern DRC provinces (Ituri, North-Kivu, South-Kivu, Maniema, Haut-Katanga, Tanganyika, Lomami, Haut-Lomami, Lualaba, Haut-Uélé and Tshopo) (Fig 1). There are three laboratories in the province of South Kivu, serving a population of 6,655,000, one laboratory in the province of North Kivu, serving around 7,574,000 people, and two laboratories in the province of Haut-Katanga, serving a population of 5,378,000 (Fig 1 and 2). Apart from these three provinces, none of the eight others provinces in eastern DRC has laboratory infrastructure capable of making a histological and/or cytological diagnosis, leaving a total population of 22,052,000 without coverage [7]. Furthermore, we note that the six laboratories identified were in urban health zones, necessitating that rural areas send samples to urban laboratories. The interviews revealed that, over the past decade, the establishment of functional exploration and endoscopy units in Lubumbashi, Bukavu, and Goma has expanded diagnostic infrastructure, encompassing both laparoscopy and endoscopy (Table 5). All the considered laboratories are integrated in hospitals except the Saturne laboratory (Bukavu) and the anatomo-cytopathology laboratory in Lubumbashi.

### Human resources human capacity

Analysis of human resources shows in five of the six laboratories the same basic composition of teams, of one pathologist surrounded by one to three histology-technicians. However, a more extensive configuration was observed at the University Clinic of Lubumbashi where two medical pathologists and medical trainees (residents specialising in the same field) worked regularly in the laboratory. In this latter laboratory, academic training is provided for medical specialization in pathology and in-depth technical training for histo-technicians, these teachings are also reinforced by a professional retraining program (transformation of laboratory technicians or medical biologists into histo-technicians) (Table 1).

**Table 1:** Indicators of structure and organisational process.

Indicators of structure and process		Sud-Kivu		North Kivu		Haut-Katanga	
**Panzi General Referal Hospital Lab**	**Bukavu Provincial General Hospital Lab**	**Saturne Lab**	**Heal Africa Lab**	**university clinics Lab**	**cyto-anatomopathology Lab**
**Human Resources**	Pathologists	1	1	1	1	2	1
	Lab technicians	3	2	2	2	3	1
**Equipment**	Optical microscope	3	2	1	2	3	1
	Digital microscope	0	0	0	0	0	0
	Embedding station	1	1	1	1	1	1
	Microtome	2	1	1	2	2	1
**Staining process**	**Standard cytological technique**						
	Papanicolaou	Yes	Yes	Yes	Yes	Yes	Yes
	MGG colouring	Yes	Yes	Yes	Yes	Yes	Yes
	**Standard histological technique**						
	Hematoxylin- Eosin	Yes	Yes	Yes	Yes	Yes	Yes
	Hematoxylin-Saffron	No	No	No	No	Yes	No
	Other staining (Shorr, Ziehl, etc.)	Yes	Yes	No	Yes	No	Yes
	**Special histological technique available**						
	Immunohistochemistry	Yes	No	No	No	No	No
	Trichrome	No	No	No	No	No	No
	Alcan Blue	Yes	No	Yes	No	No	Yes
	Other						
	**Biobanking**	Yes	Yes	Yes	Yes	Yes	Yes
	**Formol 10%**	Yes	Yes	Yes	Yes	Yes	Yes
	**Others**	No	No	No	No	No	No
**Organizational process**	Standard Operation Procedure	No	No	No	No	No	No
	Biohazard Procedure	Incomplete	Incomplete	Incomplete	Incomplete	Incomplete	Incomplete
	Computing system	No	No (Open Clinic*)	No	No	No	No
	Training	Irregular sessions	Irregular sessions	Irregular sessions	Irregular sessions	Irregular sessions	Irregular sessions
	Internal Quality Control process	Yes (irregular)	Yes (irregular)	Yes (irregular)	Yes (irregular)	No (irregular)	Yes (irregular)
	External Quality Control process	Yes (irregular)	No	No	Yes (irregular)	No	No

*OpenClinic is an online hospital data management software

The highest number of requests for analysis are recorded in the provinces of South Kivu and Haut-Katanga. In South Kivu, the laboratory of general referral hospital of Bukavu handles an annual average of 904.9 samples, while the Panzi general referral hospital’s laboratory handles 740.4 samples for a single pathologist in both laboratories. In the Haut-Katanga province, the cyto-anatomoanatomical pathology laboratory analyzed 382.7 samples for a single pathologist whereas the university clinics in Lubumbashi have an annual demand of 340.3 samples for their two pathologists (Table 3).

### Diagnostic capabilities, equipment and supplies

The diagnostic services provided by the six laboratories are relatively uniform, with basic histopathology (hematoxylin-eosin and hematoxylin-eosin Saffran staining) and cytology (Papanicolaou and May-Grünwald-Giemsa staining) widely available. Immunohistochemistry is available in one laboratory only, which is sometimes crucial for the accurate diagnosis of neoplasia, and is only accessible at the Panzi General Referral Hospital in Bukavu, South-Kivu Province, highlighting a significant gap in regional diagnostic capacity (Table 1).

Furthermore, an evaluation of essential diagnostic equipment reveals that all laboratories are equipped with the necessary apparatus for manual sample processing (e.g., microtome and embedding station) to obtain histological preparations for examination using a light optical microscope. Nevertheless, these microscopes lack cameras for image sharing and remote discussions with other pathologists (Table 1 and 5).

However, the Panzi General Referral Hospital laboratory is distinguished by its use of an automatic tissue processor and chemical protection hood. Furthermore, ancillary infrastructure, including sample storage areas and laboratory benches, is consistently present across all laboratories. Nonetheless, key equipment such as computerized specimen tracking systems, dehumidifiers, and automated histological and cytological staining machines are not universally available.

In detail, variety in equipment is observed such as: Heal Africa laboratory in North Kivu province is in possession of an Olympus optical microscope (CX21), manufactured in 2005 and acquired in 2014 (Table 4). Furthermore, the institution is equipped with a Reichert (Jung 820) manual microtome and a Viscope embedding station (Tissue TekII), both of which were acquired in 2014. Similarly, in South Kivu, the General Referral Hospital of Bukavu has an Olympus microscope (CX23) manufactured in 2012 and acquired in 2022, a semi-automatic rotary microtome (SN31243) acquired in 2022, and a BioOptica embedding station (SN2114171099) acquired in 2013. The Panzi General Referral Hospital has been equipped with an Olympus microscope (CX23) since 2022. The hospital is also equipped with a BioOptica manual microtome (300302) and a BioOptica embedding station (SN20114171099) both of which were acquired in 2013, automatic tissue processor (STP120–2, Bio-Optica chemical protection hood (SN501423056). Saturne laboratory is equipped by Olympus optical microscope (CX21), a Leitz manual rotary microtome (SN1510) and chandom embedding station (NDA). In Haut-Katanga, the University Clinic of Lubumbashi and the cytopathology laboratory are equipped with a Leica microscope (SN0495), a Leitz manual rotary microtome (SN1510), and a Chandom embedding station (NDA).

All six laboratories use 10% formalin as a fixation method prior to any histological analysis procedures. Standard histological staining techniques (Haematoxylin-Eosin and Haematoxylin-Saffron) are used in all six laboratories, while special staining techniques (Trichrome, Alcian Blue, PAS, Zhiel on tissue) are used in two laboratories only due to an irregular supply and/or a lack of reagent resources and limited funding (Tables 1,2 and 5). Papanicolaou and May Grunwald Giemsa are the only stains used for cytological diagnosis in the six laboratories in eastern DRC. All the laboratories evaluated have a tissue biobank, but none of them has a laboratory IT system or formal biobanking/tissue storage governance (Table 1).

**Table 2:** Availability of Equipment in Laboratories.

Laboratory	Optical Microscope	Microtome	Embedding Station	Automatic Tissue Processor	Chemical Protection Hood	Microscope Camera	Automated staining machine
Heal Africa (North Kivu)	Olympus CX21	Reichert Jung 820	Viscope Tissue Tek II	No	No	No	No
Bukavu General Referral Hospital (South Kivu)	Olympus CX23	Semi-automatic (2022) SN: 31243	BioOptica (2013) SN2114171099	No	No	No	No
Panzi General Referral Hospital (South Kivu)	Olympus Specific model: CX23	BioOptica (manual, 2013) Ref: 0300410 SN: 300302	BioOptica (2013) Ref:40-300-000 SN: 2114171099	Ref:STP120–2	Bioptica Ref: 50-090-002 SN: 501423056	No	No
Saturne Laboratory (South Kivu)	Olympus CX21	Leitz (manual rotatory 1510	Chandom NDA	No	No	No	No
University Clinic of Lubumbashi (Haut-Katanga)	Leica SN: 0495	Leitz (manual rotary) SN: 1510	Chandom NDA	No	No	No	No
Cytopathology Laboratory (Haut-Katanga)	Leica SN: 0495	Leitz (manual rotary) SN: 1510	Chandom NDA	No	No	No	No

### Quality assurance and control measures

The workload and sample flow vary across laboratories, with most laboratories delivering test results within an average turnaround time of seven days, although in some cases this period may extend up to six weeks Quality assurance procedures in hospitals are largely informal, as standardized operating procedures are rarely documented or systematically applied. While certain hospitals have established quality assurance units within their management structures, these units seldom engage in activities specifically targeting laboratory services. Equipment calibration, when performed, is irregular and frequently deviates from manufacturers’ recommended protocols. Accreditation from recognized laboratory bodies is limited to only a small number of hospitals, and ISO standards are rarely implemented in diagnostic practices. Consequently, compliance assessments often rely on the diagnostic results delivered to patients, which are subsequently used as the primary reference point, particularly when patients are referred to other hospitals or abroad, raising concerns about the standardization and reproducibility of diagnostic results. Participation in internal and external quality assessment programs is irregular and unsystematic, underlining the imperative of integration into established quality assurance networks in all six laboratories. Although the physical infrastructure of the laboratories is adequate in terms of space, there are significant shortcomings in terms of ventilation, with the notable exception of the Panzi General Referral Hospital laboratory. Compliance with biosafety standards is significantly compromised by inadequate ventilation. For example, fume cupboards or biosafety cabinets, which are essential for the macroscopic examination of formalin-fixed specimens, were available in only one of the six laboratories assessed. Furthermore, shortcomings in incineration facilities were observed, particularly at the Saturne Laboratory in Bukavu and the anatomo-cytopathology laboratory in Lubumbashi. The lack of personal protective equipment (PPE), including face shields, breathing masks and safety glasses, is partially regarding the different step of technical process and categories of health workers in all six laboratories, and the lack of a chemical compound to neutralize formaldehyde, apart for the Panzi General Referral Hospital laboratory, are major concerns (Table 1). These shortcomings are jeopardizing both the quality of diagnoses and the safety of staff.

### Continuing professional development

Regarding continuing professional development, the data reveals a marked disparity among laboratories. Approximately half have no system for retraining or ongoing education in areas such as diagnostic accuracy, proficiency in emerging technologies, quality assurance, data management, subspecialities, research, and biosafety, while the remainder operate irregular or inconsistent systems. This suggests significant potential for improvement in establishing regular and structured training programs. Importantly, there is no cross-sector collaboration with health and other non-governmental organizations (Tables 1 and 5).

### Laboratory workload

The data presented in Table 3 indicate marked variations in annual laboratory activity across institutions. Using laboratory registers spanning several consecutive years, average annual workloads differed by province and facility. In South-Kivu, the Panzi General Referral Hospital laboratory processed an average of 740.4 samples per year over nine years of available records, while the Bukavu Provincial Hospital laboratory handled an average of 904.9 samples annually based on thirteen years of registers, with both laboratories staffed by a single pathologist. In Haut-Katanga province, the cyto-anatomopathological laboratory analyzed an average of 382.7 samples per year over eleven years for one pathologist, whereas the University Hospital of Lubumbashi laboratory reported an annual average of 340.3 samples over the same period, distributed between two pathologists. The origin of samples indicates that the gynecology and obstetrics, surgery and internal medicine departments are the primary contributors to the laboratories of Panzi General Referral Hospital and Bukavu Provincial General Referral Hospital, which is consistent with the high overall volume of samples processed by these laboratories. Inpatient specimens also dominate, particularly for Panzi General Referral Hospital and Bukavu Provincial General Referral Hospital, while Saturne laboratory stands out for a higher number of out-of-hospital specimens. Furthermore, Panzi General Referral Hospital and Bukavu Provincial General Referral Hospital process a higher number of single biopsy procedures than the other three laboratories (Table 3).

**Table 3:** Collected Statistics: Sample Analysis, Collection Type and Subtype, and Source and Hospital Services.

Variables	South-Kivu				North-Kivu		Haut Katanga		
**Panzi General Hospital Lab**	**Bukavu Provincial Hospital Lab**	**Saturne Lab**	**Total province**	**Heal-Africa Lab**	**Total province**	**LCAP Lubumbashi**	**University hospital of Lubumbashi lab**	**Total province**
Total samples analysis	6664	11764	1836	20264	2460	2460	**4210**	3743	7953
Proportion of analyses performed (%)	94.7	92.2	100	94.3	93.2	93.2	100	100	100
Number of years of laboratory existence	11	23	10	–	10	–	17	18	–
Number of years covered by the registers found	9	13	8	–	9	–	11	11	–
Average number of samples analysis per year*	740.4	904.9	229.5	–	273.3	–	382.7	340.3	–
**Distribution of samples intra- and extra-hospital (%)**									
Samples taken from hospitalized patients within the structure housing the laboratory	5611(84.2)	11105 (94.4)	0	16717	2430 (98.8)	2430 (98.8)	0	3369 (90)	3369 (42.4)
Samples from other structures	1053 (15.8)	659 (5.6)	1836 (100)	3548	30 (1.2)	30 (1.2)	4210 (100)	373 [10]	4583 (57.6)
**Type of collection analyzed**									
Histology	4701 (70.5)	8896 (75.6)	1739 (94.7)	15336 (71.4)	2049 (83.3)	2049 (83.3)	2698 (64.1)	2438 (65.1)	5136 (64.6)
Cytology	1963 (29.5)	2868 (24.4)	97 (5.3)	4928 (28.6)	411 (16.7)	411 (16.7)	1512 (35.9)	1305 (34.9)	2817 (35.4)
**Histology**									
Proportion of simple biopsy (%)	1561 (33.2)	2918 (32.8)	598 (34.4)	5077 (33.1)	447 (21,8)	447 (21,8)	621 (23)	741 (30.4)	1362 (26.5)
Proportion of exeresis (%)	1062 (22.6)	2188 (24.6)	696 (40)	3946 (25.7)	787 (38.4)	787 (38.4)	839 (31.1)	510 (20.9)	1349 (26,3)
Proportion of Partial or total resection of an organ (%)	2078 (44.2)	3790 (42.6)	445 (25.6)	6313 (41.2)	815 (39.8)	815 (39.8)	1238 (45,1)	1187 (48.7)	2425 (47,2)
**Cytology**									
Peripheral blood smear	302 (15.4)	617 (21.5)	22 (22.7)	941 (19.1)	119(28.5)	119(28.5)	324 (21.4)	322 (24,7)	646 (22.9)
Cervicovaginal smear	1455 (74.1)	1600 (55.8)	44 (45.3)	3099 (62.9)	209 (50.8)	209 (50.8)	649 (42.9)	474 (36.3)	1123 (39.9)
Liquid puncture	206 (10.5)	614 (21.4)	22 (22.7)	842 (17.1)	74 (18.0)	74 (18.0)	323 (21.4)	187 (14.3)	510 (18.1)
Fine needle aspiration	0 (0)	0 (0)	0 (0)	0 (0)	0 (0)	0 (0)	0 (0)	0 (0)	0 (0)
Myelogram	0 (0)	37 (1.3)	9 (9.3)	46 (0.9)	9 (2.2)	9 (2.2)	216 (14.3)	231 (17.7)	447 (15.9)
Other treated samples	0 (0)	0 (0)	0 (0)	0 (0)	0 (0)	0 (0)	0 (0)	91 (7.0)	91 (3.2)
**Distribution of samples by origin (%)**									
Province housing the laboratory	6071 (91.1)	10917 (92.8)	1469 (80)	18339 (90.5)	2342 (95.2)	2342 (95.2)	4096 (97.3)	3126 (83.5)	7222 (90.8)
Neighboring provinces with pathology lab	420 (6.3)	706 (6)	367 (20)	1581 (7.8)	32 (1.3)	1 (1.3)	0 (0)	0 (0)	0 (0)
Neighboring provinces without pathology lab	113 (1.7)	71 (0.6)	0 (0)	203 (1)	71 (2.9)	0 (2.9)	114 (2.7)	430 (11.5)	544 (6.8)
Foreign countries	60 (0.9)	82 (0.7)	0 (0)	142 (0.7)	15 (0.6)	0 (0.6)	0 (0)	187 (5.0)	187 (2.4)
**Distribution of samples by hospital service (%)**									
Internal medicine service	1753 (26.3)	2612 (22.2)	263 (14.3)	4680 (23.1)	391 (15.9)	391 (15.9)	995 (23.6)	749 (20)	1744 (21,9)
Surgery service	2106 (31.6)	4188 (35.6)	437 (23.8)	6566 (32.4)	652 (26.5)	652 (26.5)	1089 (25.9)	936 (25)	2025 (25,5)
Pediatrics service	353 (5.3)	388 (3.3)	88 (4.8)	892 (4.4)	98 (4)	98 (4)	684 (16.2)	561 (15)	1245 (15,7)
Obstetrics and gynecology service	2452 (36.8)	4576 (38.9)	1048 (57.1)	8126 (40.1)	1319 (53.6)	1319 (53.6)	1442 (34.3)	1497 (40)	2939 (36,9)

*Average number of samples analysis per year = *Total samples analysis/ Number of years covered by the registers found*

**Two private laboratories *: Saturne Laboratory, Laboratoire Cyto-Anatomopathologique de Lubumbashi*

***Three Semi-State laboratories *: Panzi Hospital laboratory, Bukavu Provincial Hospital Laboratory and Heal Africa laboratory Hospital*

**** One Public or State laboratory *: Laboratory of University hospital of Lumbumbashi*

***Important note: “semi-state”*** *category reflect the specific partnership model in the DRCongo, where faith-based health structures play a major role in the public health system thanks to state contracts.*

### Service coverage and capabilities

As evidenced by the findings presented in Table 4, a total of 30,677 samples were analyzed, of which 73.4% were biopsies, while the remaining samples were of a cytological nature. In the province of South Kivu, biopsies accounted for 71.4%, while in North Kivu this figure was 83.3%. In Haut-Katanga, it was 64.6%. The proportions of simple biopsies, excisional biopsies, and partial or total organ resections varied across provinces and hospitals. However, the proportion of partial or total organ resections was higher compared to other forms of histological sampling, regardless of the province or hospital.

**Table 4:** Samples Recorded, Cyto- and Histopathological Diagnoses, and Location of Malignant Tumors in eastern DRC.

Types of collections (N = 30677)	n (%)
Histology	22521 (73.4)
Cytology	8156 (26.6)
**Diagnonis (N = 30677)**	
Malignant tumours	5546 (18.1)
Benign tumours	6122 (20)
Precancerous lesions	1011 (3.3)
Inflammatory, infectieus lesions and various pathological conditions	12630 (41.2)
Normal	5368 (17.5)
**Locations of malignant tumours (N = 5546)**	
Cervix	919 (16.6)
Prostate	782 (14.1)
Breast	773 (13.9)
Skin	306 (5.5)
Colorectal	169 (3)
Lymphoids	590 (10.6)
Others locations	2007 (36.2)

Regarding cytological samples, the cervicovaginal smear was the most frequently ordered type, representing 62.9%, 50.8%, and 39.9% in the provinces of South Kivu, North Kivu, and Haut-Katanga, respectively. This type of sample was followed by peripheral blood smears, with varying proportions across provinces. Conversely, born marrow smears were more common in Haut-Katanga, accounting for 15.9% of cytological samples, compared to 2.2% and 0.9% in the provinces of North Kivu and South Kivu, respectively. It is noteworthy that fine needle aspiration was not a procedure performed in any of the laboratories located in the eastern part of DRC.

The data shows that inflammatory lesions accounted for 41.2%, while malignant tumors represented 18.1% of all analyses conducted in these six laboratories. Among the 5,546 malignant tumors, cervical cancer ranked first (16.6%), followed by prostate, breast, and lymphoid tissue cancers (14.1%, 13.87%, and 10.66%, respectively). Colorectal cancers accounted for 3.1% of the cancers found in the six laboratories.

In most cases, the samples originated from the province where the laboratory was located: 90.5% in South Kivu, 95.2% in North Kivu, and 90.8% in Haut-Katanga. The province of South Kivu received more samples from provinces with anatomical pathology laboratories (7.8% of cases), followed by the province of North Kivu with 1.3% of cases, whereas this was rare in Katanga. The province of Katanga often received samples from laboratories without anatomical pathology labs in 6.8% of cases, with this percentage being 1% in the province of South Kivu and 2.9% in North Kivu. The laboratory in Haut-Katanga received 2.4% of samples from foreign laboratories, followed by South Kivu with 0.7% and North Kivu with 0.7%.

In all provinces, the gynecology department was the main provider of samples, with 40.1% in the province of South Kivu, 53.6% in North Kivu, and 36.9% in Haut-Katanga. This department was followed by surgery, representing 32.4% in South Kivu, 26.5% in North Kivu, and 25.5% in Haut-Katanga. Internal medicine sent approximately 23.1% of samples in South Kivu, 15.9% in North Kivu, and 21.9% in Haut-Katanga. Findings of this study show recent increase in functional exploration and endoscopy facilities in the eastern part of DRC (five in Lubumbashi, four in Bukavu and three in Goma) over the last decade

### Financial and operational constraints and patient access

Thematic analysis of the interviews indicates that the financial sustainability of laboratories relies primarily on patient out-of-pocket payments, which cover operational costs and equipment maintenance. This model results in unprocessed samples due to non-payment, underutilization of technical platforms, longer turnaround times, and limited access for rural and low-income populations, leading to delayed diagnoses, treatment postponements, and abandonment of follow-up care (Tables 1 and 5)

**Table 5:** Thematic Analysis of Laboratory Functioning, Capacity, and Patient Impact.

Domain	Main Themes	Sub-Themes	Illustrative Quotes
Infrastructure and Equipment	Insufficient equipment	Obsolete materials	*Quote 1: “At our laboratory, the equipment has been in use for over ten years. While it still functions, it no longer meets the needs of modern diagnostic techniques (…)” Quote 2: “…We often have to improvise with old equipment to carry out some analyses (…)” Quote 3: “…At this laboratory, the lack of a camera on the microscope limits sharing images with other pathologists (…)”*
		Lack of modernization	*Quote 1: “In our laboratory, most procedures remain manual. We do not have an automated tissue processor or automated staining machines (…)” Quote 2: “…We do not have any electronic system to manage sample tracking and patient data (…)”*
	Maintenance and calibration	Lack of technical follow-up	*Quote 1: “…We do not have a regular maintenance plan for our instruments (…)”* *Quote 2: “At our laboratory, equipment calibration is rarely performed, often only when a device breaks down (…)”*
	Reagent and consumables	Frequent stockouts	*Quote 1: “In our lab, some special stains are never performed due to lack of supplies (…)” Quote 2: “…We have to adjust our techniques based on available reagent and consumables, which affects the quality of results (…)” Quote 3: “…Sometimes we have to suspend tests for several weeks due to reagent shortages (…)”*
	Expansion of Diagnostic Facilities	Increase in functional exploration and endoscopy units	*Quote 1 (Lubumbashi): “Over the last decade, five new functional exploration and endoscopy units have been established in Lubumbashi, contributing to a higher number of samples received from gynecology and internal medicine.”* *Quote 2 (Bukavu): “In Bukavu, four new centers now provide additional exploration capacity with endoscopy and laparoscopy without improving pathology capacity, which has steadily increased sample submissions.”* *Quote 3 (Goma): “Three new facilities in Goma have enhanced local diagnostic infrastructure, resulting in more samples being sent to our laboratory.”*
Human Resources	Shortage of specialists	Few pathologists and technicians	*Quote 1: “In our province, there is often only one pathologist for the entire population (…)” Quote 2: “…We have very few specialized technicians, which slows down sample processing (…)”*
	Training	Lack of continuing education	*Quote 1: “At this laboratory, refresher training is rarely organized (…)” Quote 2: “…We have never received training on modern cytology or immunohistochemistry techniques (…)” Quote 3: “…Staff mostly improve on the job, with minimal academic supervision (…)”*
	Workload	Fragmented and variable workload	*Quote 1: “In our laboratory, samples arrive irregularly, forcing us to batch analyses, which delays results (…)” Quote 2: “…We have periods of high workload and others with almost nothing to process (…)”*
Diagnostic Capabilities	Limited advanced techniques	Immunohistochemistry	*Quote 1: “Here, immunohistochemistry is only possible for very specific cases and often requires sending samples elsewhere (…)” Quote 2: “…We lack the equipment, training, and reagents for some complex analyses (…)”*
Procedures and Quality Assurance	Protocols	Lack of SOPs and standardization	*Quote 1: “At this laboratory, everyone follows their own habits for analysis (…)” Quote 2: “…We do not have written standard procedures, which complicates result comparison (…)”*
	Quality control	Weak internal quality assurance	*Quote 1: “Even though we have a quality unit, it is not actively involved in daily processes (…)” Quote 2: “…At this laboratory, quality control often relies on samples sent to other laboratories for confirmation (…)”*
Accessibility and Logistics	Geographic distribution	Urban concentration	*Quote 1: “Patients from rural areas must travel hundreds of kilometers to submit a sample (…)” Quote 2: “…With most laboratories situated in cities, patients from rural areas struggle to reach the facility, causing numerous samples to go unprocessed (…)”*
	Sample transport	Poor conservation	*Quote 1: “We sometimes receive biopsies in poor condition due to inadequate transport or fixation (…)” Quote 2: “…At this laboratory, the quality of samples depends heavily on the journey from the villages or other cities (…)”*
	Delays	Long turnaround times	*Quote 1: “At this laboratory, some results take up to six weeks, especially for complex tests (…)” Quote 2: “…Delays at this facility can postpone the start of patient treatment (…)”*
Financing and Governance	Financial resources	Low public funding	*Quote 1: “We operate almost entirely on patient fees, without significant government support (…)” Quote 2: “…At this laboratory, equipment maintenance relies on the income generated from analyses (…)”*
	Cost for patients	High costs	*Quote 1: “Tests are often unaffordable for most patients (…)” Quote 2: “…We see families abandon care due to financial constraints (…)”*
	Health policy	Absence of national plan	*Quote 1: “... there is no national plan guiding the development of cancer diagnostics (…)”*
Impact on Patient Care	Diagnostic delays	Late presentation	*Quote 1: “Many patients arrive at advanced stages of disease at this hospital (…)” Quote 2: “…We frequently see cancers diagnosed too late, limiting treatment options (…)”*
	External dependence	Sending samples abroad	*Quote 1: “For certain specialized analyses, we have to send samples abroad (…)” Quote 2: “…At this laboratory, this results in extra costs and delays for patients (…)”*
	Social consequences	Abandonment of follow-up	*Quote 1: “We observe some families abandoning medical follow-up due to time for results and high fees (…)”* *Quote 2: “…Time for results and high fees in the laboratory discourage patients from returning for results or treatment (…)”*

## Discussion

The study highlights marked disparities in the diagnostic capacity of pathology laboratories in Eastern Democratic Republic of Congo, laboratories are concentrated in only three of the eleven provinces, leaving more than 22 million inhabitants without access to cancer diagnostic services, with only six laboratories concentrated in urban areas. Human resources are often restricted to a single pathologist and histotechnician per facility. Diagnostic capacity remains limited, with immunohistochemistry available in only one center and a notable absence of fine-needle aspiration cytology, digital equipment such as microscopes with cameras and slide scanners for telepathology, and standardized protocols. These gaps restrict access to expert opinions, hinder opportunities for collaborative diagnostic review, and undermine both diagnostic accuracy and therapeutic decision-making. In terms of case distribution, inflammatory and infectious lesions are predominated, followed by benign and malignant tumors, the latter mainly represented by cancers of the cervix, prostate, breast and colorectal cancer. The lack of structured quality assurance and continuous training further illustrates the fragility of the system and underscores the urgent need to strengthen local capacities and underline the imperative of integration into established quality assurance networks in all six laboratories. In addition, gaps in continuing education consisting of diagnostic accuracy, proficiency in emerging technologies, quality assurance, data management, subspecialties, research, and biosafety may compromise the reliability of results and the overall effectiveness of services. Context-appropriate solutions, including hands-on workshops, telepathology, e-learning, mentorship, and partnerships with reference centers, have the potential to sustainably strengthen professional competencies and enhance diagnostic performance

Recent epidemiological studies on the African continent reveal high prevalence and incidence rates for cervical cancer, in contrast to industrialized countries where screening programs have significantly reduced these same rates, complemented by mass HPV vaccination programs. Furthermore, colorectal cancer is gradually emerging as a major public health problem for African populations, whereas it was once considered a health concern mainly of industrialized countries. This development is in line with our observations and those reported by other researchers [23,24]. Findings show that only 26.6% of analyses carried out in the 6 evaluated pathology laboratories are cytological. This trend is corroborated by research carried out in Nigeria [25], where cytological analyses accounted for 27.5% of laboratory studies. In contrast, in industrialized countries such as the United States, cytological tests account for 40% of laboratory studies [26]. This discrepancy can be explained by the fact that cytological techniques are only established in a few and scattered locations, whereas the need for this type of service is considerable on the African continent.

The results of this study highlight the preponderance of inflammatory pathologies (which include infectious tissue diseases and other inflammatory conditions, including tuberculous (TB), parasitic, fungal and non-specific inflammatory processes), which accounted for 41.1% of the analyses performed in the six laboratories surveyed While malignant and benign tumours represent respectively 18,1% and 20%

These data are like those reported by Berezowska and colleagues [27] in Malawi, where non-tumoral conditions accounted for 47.4%, with a predominance of inflammatory conditions, while malignant tumours accounted for 4.1%. Similarly, the study carried out by Ismail et al. in Mozambique [5] revealed a prevalence of 22.2% for inflammatory conditions. The variation in the prevalence of cancerous lesions goes in the same direction as the general upward trend in cancer prevalence and incidence on the African continent, as shown by Berezowska’s study in 2012, Ismail’s study in 2021 and our own study in 2023 [5,27].

Except for the Saturne laboratory and the anatomocythopathology laboratory in Lubumbashi, the laboratories studied are integrated into hospitals. The volume of work carried out in these laboratories highlights the importance of anatomical pathology diagnosis in routine clinical practice. Conversely, anatomical pathology laboratory that are not part of hospitals or health establishments receive fewer samples. Indeed, as has been demonstrated in other disciplines of laboratory medicine, such as clinical microbiology, close collaboration between laboratory specialists and clinicians is the cornerstone of appropriate patient management [28].

The Department of Gynaecology and Obstetrics is the primary contributor of samples to the pathology laboratories, with the Departments of Surgery and Internal Medicine following closely behind. This trend is partly explained by the need for anatomicalpathology analysis for many gynaecological pathologies and obstetric complications. Furthermore, the tendency for consultations to occur at a later stage in the patient’s illness has been observed to result in a greater frequency of surgical samples and a higher number of cases involving partial or total organ removal when compared to other sampling methods. This study shows recent increase in functional exploration and endoscopy facilities in the eastern part of DRC over the last decade could also be a contributing factor to the high number of samples from internal medicine in this region. Similar observations were reported in a study conducted in Malawi [27]. The noteworthy frequency of simple biopsies and biopsy excisions, frequently observed in the laboratories of the Panzi General Referral Hospital, the Bukavu Provincial General Referral Hospital and the Heal Africa Hospital, could be attributed to the enhanced technical facilities available in these institutions, including diagnostic and interventional gastroenterology, minimally invasive surgery, oncology surgery, as well as patient awareness of screening or early diagnosis by specialist physicians.

As far as cytological analyses are concerned, cervico-vaginal smears and peripheral blood smears are routinely performed in all six laboratories. However, mass puncture cytology, fine needle aspiration cytology, urinary cytology and bronchoalveolar lavage cytology are less frequently performed. Even if fine-needle aspiration cytology is often considered as an economical, simple and rapid method of cytological diagnosis, it requires a skilled cytopathologist, preferably on site. Moreover, cytology often leaves several cases with a suspicious diagnosis of malignancy without a definitive diagnosis, and a significant proportion of samples unsuitable for diagnosis which represent two major drawbacks [29–32].

While the literature shows that specialised training programmes and the establishment of a diagnostic consultation framework in the form of a network between cancer diagnostic laboratories both within and outside the country are essential to ensure (i) the standardisation and quality of diagnostic procedures, (ii) the improvement of staff skills, and (iii) epidemiological surveillance, our study reveals the absence of continuous training (or the presence of irregular continuous training) for staff and the lack of formal consultation among pathologists [33–35].

Our study reveals a concentration of laboratories in urban areas, demonstrating the efforts made to optimise access to specialised diagnostic services in densely populated zones. This finding can be explained by the recurrent conflicts and prevailing insecurity in rural areas, combined with insufficient financial resources and a decrease in rural population due to displacement. These factors constitute major obstacles to the establishment of local laboratories in these regions. Additionally, health facilities in villages are often forced to send samples or patients in urban areas, to laboratories or hospitals offering the necessary diagnostic capabilities. The geographical inaccessibility of urban laboratories, due to the state of the country’s road infrastructure, also poses significant logistical constraints. These factors have contributed to a regression in achieving developmental milestones for cancer diagnostics [14,36,37]. However, beyond the evident gaps in infrastructure and geographical coverage, our findings reveal a critical and often overlooked opportunity: the significant underutilization of existing technical capacity. As detailed in Section three of the results, laboratories like Panzi General Referral Hospital operate far below their potential throughput. This underutilization is primarily driven by non-technical barriers such as fragmented sample transport systems, a lack of clinician awareness in peripheral areas regarding available services, and pervasive financial constraints that limit patients’ ability to pay for diagnostics and labs’ ability to procure consumables. Therefore, a strategic shift in focus is warranted. Rather than solely prioritizing the construction of new laboratories—a capital-intensive and long-term endeavour immediate gains in diagnostic access could be achieved by optimizing current resources. Investing in enhanced logistics, targeted training for clinicians on the value of pathology, and deploying telepathology networks could dramatically increase testing volumes without the need for new physical infrastructure.

Crucially, the nature of the equipment gaps identified must be precisely understood. The absence of advanced technology such as high-resolution microscopes with cameras, whole-slide scanners, does not fundamentally paralyze current basic service provision. Instead, these gaps constitute a significant impediment to diagnostic reliability, turnaround time, and the expansion of service offerings towards more complex diagnoses (via advanced immunohistochemistry or molecular techniques). This distinction suggests a two-tiered strategic approach: initial investments should first target overcoming the operational and logistical barriers to fully utilize existing capacity, while subsequent investments can strategically upgrade technological capacity to improve quality and expand services.

Consequently, any future interventions must also address the foundational financial models and quality assurance frameworks. According to the interviews, the financial viability of the operations is ensured by the income generated from analyses paid by the patients without medical insurance, which also covers the maintenance and renewal of the equipments, this situation leads to a considerable number of samples remaining unprocessed due to non-payment of laboratory fees. Consequently, the prevalence of cancer is likely underestimated, the existing technical platforms are underutilized, and the turnaround time for delivering results is adversely affected. Moreover, the high costs imposed on patients significantly limit access for rural and low-income populations, resulting in delayed diagnosis, postponed treatment, and, in some cases, the abandonment of follow-up care.

Resolving this issue need ensuring financial sustainability through partnerships and hybrid models that combine local revenue with external support. When integrated into a coordinated regional strategy, these measures could substantially enhance access, timeliness, and diagnostic accuracy. Lastly, the lack of financial autonomy and limited access to education for women delay or prevent their access to cancer diagnostic and treatment services. Studies highlight the importance of awareness programmes to improve women’s knowledge as a key element in enhancing access to cancer screening and diagnosis. Moreover, it is essential to address cancers affecting all genders, including prostate cancer and colorectal cancer, in order to promote gender inclusivity [14,33,34].

## Conclusion

The DRC is one of the largest countries in central Africa with an estimated population of more 100 million habitants. The near continuous internal conflicts of the last three decades have left the healthcare system in a very fragile state, and little information exists for current capacities. This is particularly important for cancer diagnosis and treatment, as an upward trend is evident at a population level across the entirety of the African continent. This in-depth analysis of pathology laboratories in eastern DRC highlights major challenges in terms of infrastructure, diagnostic capacity, quality and accessibility of services. It is imperative to mobilize concerted efforts to improve diagnostic health systems, with an emphasis on formalising quality protocols, widening access to specialized services and involving local communities in the fight against cancer.
